# Resistance to Chytridiomycosis in European Plethodontid Salamanders of the Genus *Speleomantes*

**DOI:** 10.1371/journal.pone.0063639

**Published:** 2013-05-20

**Authors:** Frank Pasmans, Pascale Van Rooij, Mark Blooi, Giulia Tessa, Sergé Bogaerts, Giuseppe Sotgiu, Trenton W. J. Garner, Matthew C. Fisher, Benedikt R. Schmidt, Tonnie Woeltjes, Wouter Beukema, Stefano Bovero, Connie Adriaensen, Fabrizio Oneto, Dario Ottonello, An Martel, Sebastiano Salvidio

**Affiliations:** 1 Department of Pathology, Bacteriology and Avian Diseases, Faculty of Veterinary Medicine, Ghent University, Merelbeke, Belgium; 2 Dipartimento di Scienze della Vita e Biologia dei Sistemi, Università degli Studi di Torino, Torino, Italy; 3 Zirichiltaggi – Sardinia Wildlife Conservation, Sassari, Italy; 4 Waalre, The Netherlands; 5 Institute of Zoology, Zoological Society of London, London, United Kingdom; 6 Department of Infectious Disease Epidemiology, Imperial College, School of Public Health, London, United Kingdom; 7 Institut für Evolutionsbiologie und Umweltwissenschaften, Universität Zürich, Zürich, Switzerland; 8 Koordinationsstelle für Amphibien- und Reptilienschutz in der Schweiz, Neuchâtel, Switzerland; 9 Stichting Reptielen, Amfibieën en Vissen Onderzoek Nederland, Nijmegen, The Netherlands; 10 Faculty of Geo-Information Science and Earth Observation, University of Twente, Enschede, The Netherlands; 11 Dipartimento di Scienze della Terra, dell’Ambiente e della Vita, Università di Genova, Genova, Italia; Leibniz Institute for Natural Products Research and Infection Biology- Hans Knoell Institute, Germany

## Abstract

North America and the neotropics harbor nearly all species of plethodontid salamanders. In contrast, this family of caudate amphibians is represented in Europe and Asia by two genera, *Speleomantes* and *Karsenia*, which are confined to small geographic ranges. Compared to neotropical and North American plethodontids, mortality attributed to chytridiomycosis caused by *Batrachochytrium dendrobatidis* (*Bd*) has not been reported for European plethodontids, despite the established presence of *Bd* in their geographic distribution. We determined the extent to which *Bd* is present in populations of all eight species of European *Speleomantes* and show that *Bd* was undetectable in 921 skin swabs. We then compared the susceptibility of one of these species, *Speleomantes strinatii*, to experimental infection with a highly virulent isolate of *Bd* (*Bd*GPL), and compared this to the susceptible species *Alytes muletensis*. Whereas the inoculated *A. muletensis* developed increasing *Bd*-loads over a 4-week period, none of five exposed *S. strinatii* were colonized by *Bd* beyond 2 weeks post inoculation. Finally, we determined the extent to which skin secretions of *Speleomantes* species are capable of killing *Bd*. Skin secretions of seven *Speleomantes* species showed pronounced killing activity against *Bd* over 24 hours. In conclusion, the absence of *Bd* in *Speleomantes* combined with resistance to experimental chytridiomycosis and highly efficient skin defenses indicate that the genus *Speleomantes* is a taxon unlikely to decline due to *Bd*.

## Introduction

With more than 430 species, the family Plethodontidae comprises the majority of extant urodelan species and has experienced a marked evolutionary radiation in North, Central and northern South America [1]. In the rest of the world, this family is confined to the Maritime Alps, the central Apennine mountains in continental Italy and Sardinia in Europe (the genus *Speleomantes*, containing 8 species), and to South Korea (1 species, *Karsenia koreana*). The European plethodontids are closely related to the North American genus *Hydromantes* [1] and occupy an area well known to be infected by the amphibian pathogenic fungus *Batrachochytrium dendrobatidis* (*Bd*), one of the known drivers underlying global amphibian declines [2]–[6]. In Italy and France, the two countries where the genus *Speleomantes* occurs, the aggressive lineage of the pathogen, the *Bd* global panzootic lineage (*Bd*GPL), also occurs [7]. Both infection and mortality due to *Bd* has been reported for both countries, including localities where *Speleomantes sp.* are endemic [3], [4], [6], [8]. Although habitat alteration has taken its toll on *Speleomantes* populations, enigmatic declines that would match chytridiomycosis driven declines witnessed elsewhere have not been reported. Indeed, these salamanders are among the most abundant vertebrates in suitable habitats [9]. The skin is of vital importance to plethodontid salamanders, which rely exclusively on cutaneous respiration. Chytridiomycosis dramatically disturbs the skin function [10], [11] and thus compromises respiration. Therefore, chytrid infections in *Speleomantes* should result in rapid killing of the plethodontid host, as has been hypothesized to be the case for several declining neotropical plethodontids and demonstrated in some, but not all [12], North American species [13]–[16]. Although different infection protocols have been used in these studies, they clearly demonstrate that some New World plethodontid species are easily colonized by the fungus.

Hitherto no suspected chytridiomycosis associated declines in *Speleomantes* have been observed, even in a region where chytridiomycosis occurs. This leads us to hypothesize first that prevalence of lethal cutaneous infections, such as infections by *Bd*, are low in European plethodontid salamanders. For this purpose, we determined to what extent *Bd* is present in populations of all eight species of *Speleomantes*.

Susceptibility to clinical chytridiomycosis, however, varies greatly among plethodontid species. In amphibian hosts, differences in host susceptibility have been attributed to the presence of fungicidal skin microbiota [17]–[19], antimicrobial peptides (reviewed in Rollins-Smith [20]), host genetics [21]–[23] and/or environmental factors (e.g. [8]), which may affect invasion of amphibian skin by the pathogen [24]. This leads us to hypothesize that resistance of European plethodontid salamanders is due to skin defenses that efficiently cope with *Bd* infection. Subsequently, we then examined susceptibility of *Speleomantes strinatii* to experimental infection with a global panzootic lineage strain of *Bd* (*Bd*GPL isolate IA2011, [7]). Finally, we determined to what extent skin secretions of *Speleomantes* species are capable of killing *Bd*.

## Materials and Methods

All animal experiments were conducted according to biosecurity and ethical guidelines and approved by the ethical committee of the Faculty of Veterinary Medicine, Ghent University (EC2011-073). All species involved in this study are protected as defined in Annex IV of the EU Habitats Directive (Council Directive 92/43/EEC on the Conservation of natural habitats and of wild fauna and flora). The entire experiment was submitted and approved by the Italian Ministry of Environment that issued permits to SS (issue numbers: DPN-2010-0010807 and PNM-2012-0007331). In Italy, state permits are valid over the entire country, since wildlife is a public property (national law 157/92). In addition however, when salamanders were sampled inside Protected Areas, local permits were also obtained from the “Parco Regionale Frasassi and Gola della Rossa” (permit number 3774/2012) the “Parco Regionale delle Alpi Apuane” (permit number DD.5/2012) and “Parco Regionale Alpi Marittime” (permit DD.165a/2011 issued to DO and FO). Permit PNM-2012-0007331 was also valid to capture animals (outside Protected Areas) to be used in experimental infections at Ghent University (EC2011-073). Since the study did not involve work on living animals in Italian laboratories, authorisation from the Italian Ministry of Health was not required.

**Figure 1:**
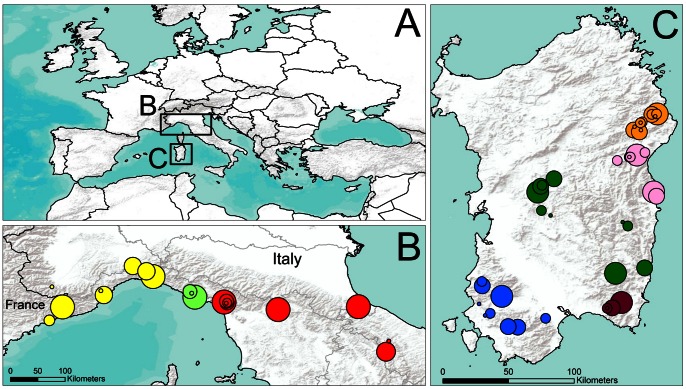
Sampling locations for *Bd* in Europe. The boxed areas in the larger map of Europe (**A**) show the geographic locations. Expanded maps show the collection sites in southeastern France, mainland Italy (**B**) and Sardinia (**C**). For map (**B**), the colours represent the species *Speleomantes strinatii* (yellow), *S. ambrosii* (bright green) and *S. italicus* (red). For map (**C**), the colours represent the species *S. genei* (blue), *S. sarrabusensis* (purple), *S. imperialis* (dark green), *S. supramontis* (pink) and *S. flavus* (orange). Localities are indicated by symbols proportional to sample size.

Between December 2004 and September 2012, we sampled 921 specimens including examples of all 8 recognized species of *Speleomantes* (*Speleomantes ambrosii*, *S. flavus*, *S. genei*, *S. imperialis*, *S. italicus*, *S. sarrabusensis*, *S. strinatii*, *S. supramontis*) at 65 localities in mainland Italy and southern France (351 samples) and Sardinia (570 samples) (**Fig. 1** **, Table S1**). Samples were collected by rubbing the abdomen, feet and the ventral side of the tail at least 10 times as has been described by Van Rooij et al. [25] using a rayon tipped swab (160 C, Copan Italia S.p.A., Brescia, Italy). The sex of the adults was recorded for the three continental species (*S. italicus*, *S. ambrosii* and *S. strinatii*) by checking for the presence of the typical male mental gland [9] and the ratio females: males: juveniles was approximately 1∶1∶1. DNA from the swabs was extracted in 100 µl PrepMan Ultra (Applied Biosystems, Foster City, CA, USA), according to Hyatt et al. [26]. DNA samples were diluted 1∶10 and quantitative PCR (qPCR) assays were performed in duplicate on a CFX96 Real Time System (BioRad Laboratories, Hercules, CA, USA). Amplification conditions, primer and probe concentrations were according to Boyle et al. [27]. Within each assay, 1 positive control sample containing *Bd* DNA from a naturally infected and deceased Costa Rican *Eleutherodactylus* sp. as template and 3 negative control samples with HPLC water as template were included. Samples were considered positive for *Bd* when a clear log-linear amplification was observed, when the number of genomic equivalents (GE) of *Bd*, defined as the measure of infection, was higher than the detection limit of 0.1 GE and when amplification that met both of the previous criteria was observed in both replicates. In case of conflict between both replicates of the same sample, the sample was run again in duplicate. To control and estimate inhibition, a subset of samples negative for the presence of *Bd* (n = 84) was retested under the same conditions as described above, but with an exogenous internal positive control (VIC™ probe, Life technologies, Austin, TX, USA) included as described by Hyatt et al. [26]. The Bayesian 95% credible interval for prevalence was estimated as described by Lötters et al. [28].

We assayed susceptibility to chytridiomycosis in five male subadult *S. strinatii* by experimentally exposing them to a controlled dose of *Bd*. Ten captive bred juvenile *Alytes muletensis* were used as susceptible, positive control animals [29]. The animals were housed individually in plastic boxes (20×10×10 cm) lined with moist tissue, provided with PVC-tubes as shelter and kept at 18°C. Crickets were provided as food items *ad libitum*. All animals were sampled for the presence of *Bd* before inoculation using the method described above. A virulent isolate of the global panzootic lineage of *Bd* (*Bd*GPL IA2011) [7], isolated in 2011 in the Spanish Pyrenees and capable of causing severe chytridiomycosis in urodelans was used in this study. All animals were exposed to a single dose of 1 ml of distilled water containing 10^5^ zoospores/ml. Skin swabs were collected weekly for 4 weeks and processed as described above to determine the infection load for *Bd* each animal exhibited over the course of the experiment. After termination of the experiment, all animals were treated with voriconazole [30]. The *S. strinatii* specimens are still kept following strict biosecurity guidelines at the clinic for Exotic Animals and Avian Diseases (Faculty of Veterinary Medicine, Ghent University) for further follow-up.

To determine the extent to which skin secretions of *Speleomantes* are capable of killing *Bd* zoospores, skin secretions were collected non-invasively from wild individuals of 7 of the 8 *Speleomantes* species (**Table S2**) and processed within 1 h (*S. strinatii*), 48 h (*S. ambrosii, S. italicus, S. flavus, S. supramontis, S. genei*) or 72 h (*S. sarrabusensis*). For this purpose, a microbiological inoculating loop was gently rubbed over the dorsal tail until white skin secretions accumulated on the loop. Prior to sampling sterile loops were cut off, stored individually in sterile vials and the total weight of each vial was determined. Collected skin secretions were weighed to the nearest 0.1 mg by subtracting the weight of the vial and the inoculation loop from the total weight. Varying amounts of skin secretions were collected per *Speleomantes* specimen, ranging from 3.1 to 32.7 mg. Collected skin secretions were not further diluted prior exposure to *Bd* zoospores. Loops with secretions were incubated in a zoospore suspension. To keep the ratio between the amount of skin secretions and *Bd* zoospores added constant, 10 µl of zoospore suspension containing 10^6^ zoospores/ml distilled water was added per mg skin secretion. Samples were incubated for 24 h at 20°C. At 0 and 24 h of incubation, the number of viable zoospores was assessed using qPCR on the zoospores that were pretreated with ethidium monoazide (EMA, Sigma-Aldrich Inc., Bornem, Belgium) as described by and validated in Blooi et al. [31]. Viable/death differentiation is obtained by covalent binding of EMA to DNA in dead *Bd* by photoactivation. EMA penetrates only dead *Bd* with compromised membranes and DNA covalently bound to EMA cannot be PCR amplified [32]. In brief, at 0 and 24 h of incubation a 5 µl aliquot was taken from each sample, transferred into a 24-well plate and 195 µl TGhL broth (tryptone, gelatin hydrolysate, lactose) was added to each well for its protective effect on viable *Bd* zoospores during EMA treatment. Negative controls for skin secretion activity consisted of zoospore suspensions not exposed to skin secretions in order to quantify the ‘natural’ loss of viability in *Bd* zoospores, while positive controls were heat-killed zoospores. Five µl of a 1 mg/ml stock solution of EMA in dimethyl formamide was added to 200 µl zoospore suspension in TGhL broth to obtain a final concentration of 25 µg/ml EMA, incubated for 10 minutes, protected from light and exposed to a 500 W halogen light at 20 cm distance for 5 minutes. Then, samples were washed by centrifugation (5000 rpm, 5 min, 20°C), the supernatant was discarded and the pellet was suspended in HPLC water. In parallel, the total amount of *Bd* zoospores in all samples, including controls, was enumerated using exactly the same procedure as described above, only 5 µl HPLC water was added to each sample instead of EMA. DNA extraction and qPCR were then done as described above. Killing activity was expressed as log(10) reduction of viable spores in a given sample compared to the negative controls.

## Results

None of the 921 skin swabs collected from any *Speleomantes* sp. tested positive for *Bd*. The Bayesian 95% credible interval for the observed prevalence of 0% is (0.0000, 0.0040). However, in 12 out of 84 samples tested (14%) PCR-inhibition occurred that could not be abolished by diluting the samples 1/100. Extrapolated to the total number of individual salamanders tested, this reduces the reliable number of *Bd*-negative salamanders to 789 with a corresponding 95% credible interval of (0.0000, 0.0047).

**Figure 2:**
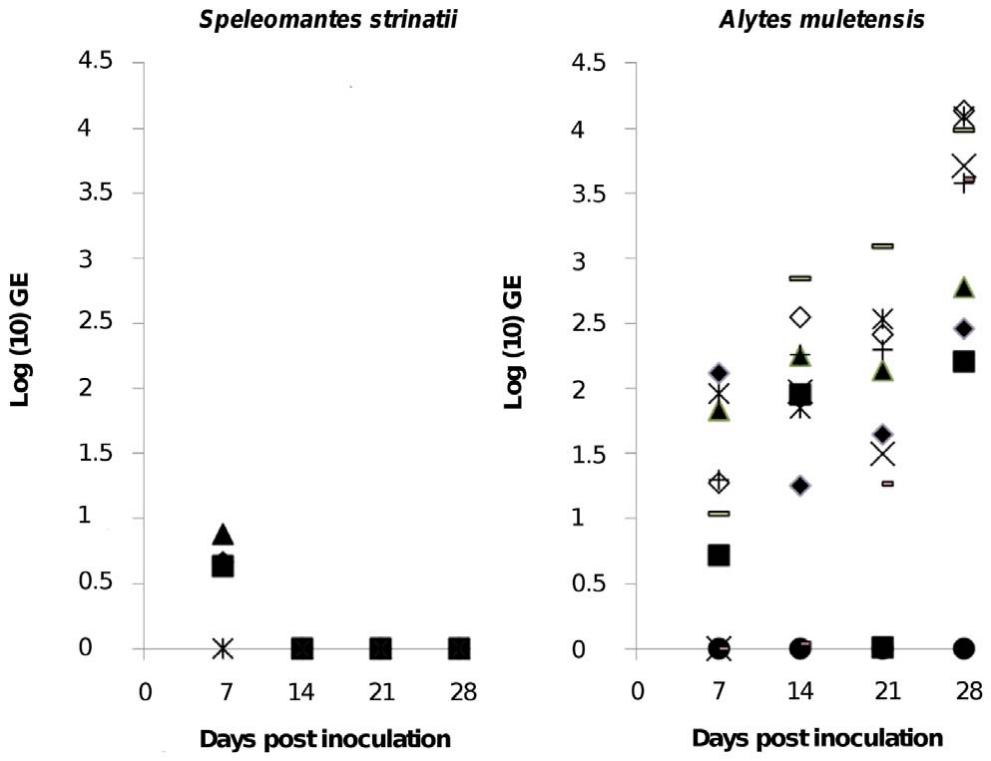
Experimental infection of *Speleomantes strinatii* with *Bd*. Infection loads are represented as log (10) genomic equivalents (GE) of *Bd* in skin swabs from *Speleomantes strinatii* (left panel) and compared with *Alytes muletensis* (right panel) serving as positive control animals, up to four weeks post experimental inoculation with *Bd*. Each symbol represents an individual animal.

None of the experimental animals tested positive for *Bd* prior to the exposure. Over the four week infection period, 9/10 *Alytes muletensis* developed marked infection with increasing *Bd* loads. Of the 5 inoculated *Speleomantes strinatii*, 3 animals exhibited weak infections (small GE value) at 7 days post infection (dpi) with an average of 5.5±1.8 GE per swab. At 14 dpi, one salamander was borderline positive (0.2 GE per swab) but at 21 and 28 dpi all salamanders tested negative for *Bd*. In contrast, *A. muletensis* exhibited median GE counts of 91 at 21 dpi and 3920 at 28 dpi (**Fig. 2**). No clinical signs were noticed in the infected salamanders. For animal welfare reasons, *A. muletensis* were treated at 4 weeks pi using voriconazole to clear infection, as described by Martel et al. [30].

**Figure 3:**
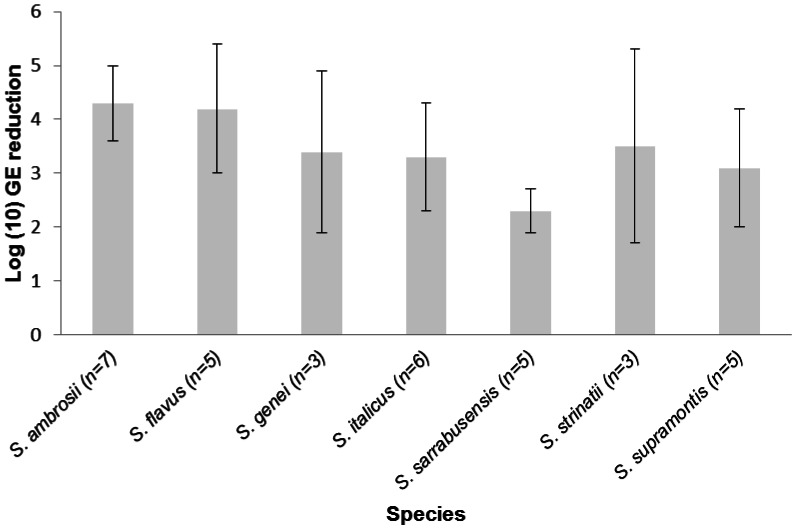
Killing activity of skin secretions of *Speleomantes* species against *Bd*. Killing activity of *Speleomantes* skin secretions at physiological concentrations is expressed as log(10) viable spores of *Bd* added to the skin secretions –log(10) viable spores recovered 24 h later. Results are presented as mean genomic equivalents of *Bd* (GE) ± standard error (SEM); n = sample size.

Skin secretions of all *Speleomantes* species were capable of efficiently killing *Bd* zoospores (**Fig. 3**). Exposure to skin secretions resulted in a 200 to 20000 fold reduction of the number of viable spores within 24 h post exposure.

## Discussion

*Bd* infections appear to be highly uncommon, if not absent, in adult and juvenile *Speleomantes* of all species and throughout their range. This observation is strengthened by the recent publication of Chiari et al. [33], reporting absence of *Bd* in Sardanian *S. flavus*, *S. genei*, *S. imperialis*, *S. sarrabusensis* and *S. supramontis* (n = 143). The genus *Speleomantes* occupies an ecological niche highly suitable to *Bd* colonization, persistence and spread due to relatively low preferred body temperatures (<18.5°C, reviewed by Lanza et al. [9]) and higher humidity environments. Contacts that would facilitate interspecific transmission are also common because at some locations salamanders occur outside of caves and are found under retreat sites with other species that are capable of carrying infections (FP personal observations, see also [3], [4], [6]). Intraspecific transmission would also be highly likely, as courtship involves intimate contact, salamanders crowd together in summer retreats, and juveniles also exhibit highly aggregated distribution patterns at certain times of the year [34]. Thus, substantial opportunity exists for *Bd* for introduction into *Speleomantes* populations and to amplify rapidly once introduced, but neither of these seems to have occurred to any significant degree. Field studies of other amphibian species have reported low prevalence or absence of detectable infection in species that show seasonal fluctuations in prevalence 35–37. We find this an unlikely explanation for the observed absence of infection. Moreover, for the present study samples were taken from December to September and over multiple years. Seasonally mediated fluctuations are associated with strong variation in environmental metrics that influence *Bd* growth and reproduction [35], [36], [38] suggesting that more stable environments should result in more consistent patterns of infection (see [39]). However, recent evidence suggests that variable temperatures may be most favorable for *Bd* driven declines, probably due to temperature drop induced zoospore release [40]. Variation of prevalence should be minimized in the cave-dwelling *Speleomantes sp.*, where relatively stable cave temperatures and moisture regimes should buffer against environmentally mediated changes in prevalence. Further, our experimental results indicate the infection is unlikely in at least one species and the majority of *Speleomantes* species are equipped with the tools to resist infection.

Exposing *S. strinatii* to a highly virulent *Bd* strain and in a manner that resulted in potentially lethal infection in a susceptible host did not result in persistent infection of the salamanders. It is possible that the low GE values detected until two weeks post inoculation in *S. strinatii* represent dead *Bd* cells or some form of *Bd* DNA contamination rather than active infection. Thus it is possible that *S. strinatii* is extremely efficient at blocking epidermal colonization by *Bd* even when exposed to a highly concentrated and strong dose of *Bd* zoospores, but our experimental design prevents us from distinguishing between this and rapid clearing of infection. Since successful epidermal colonization by *Bd* requires keratinocyte invasion [22], [41], [42], we hypothesized that *Speleomantes* skin contains highly effective fungicidal properties that prevent skin invasion. Indeed, we showed that *Speleomantes* skin secretions were very efficient in killing *Bd* zoospores as assessed using a recently developed and highly reproducible assay [31]. Factors present in the skin secretions that account for the observed *Bd* killing need further identification but probably include antimicrobial peptides (AMP) [43]–[45] and/or bacterially produced metabolites [17], [18], [46]. AMPs that play a defensive role against invasion by pathogenic microorganisms have been described for other Ambystomidae and plethodontid species [47]–[49] but not characterized. Hitherto, only the antifungal metabolites 2,4-diacetylphloroglucinol, indol-3-carboxaldehyde and violacein have been identified that are secreted by symbiotic bacteria residing on the skin of plethodontid species *Plethodon cinereus* and *Hemidactylium scutatum* [17], [46]. Moreover, these metabolites may work synergistically with AMPs to inhibit colonization of the skin by *Bd* [50]. Further characterization of such AMPs and the composition of microbial skin communities, combined with the study of their assessment in plethodontid species and/or populations can open new perspectives for further understanding factors mediating resistance towards chytridiomycosis, its control and mitigation. In addition, a study of microbial skin communities has the potential to direct probiotic conservation strategies for susceptible species in the area.

The apparent absence of *Bd* and chytridiomycosis driven declines in *Speleomantes* throughout their range, lack of colonization or sustained infection in experimentally infected animals and pronounced *Bd* killing capacity of *Speleomantes* skin secretions together suggest the genus *Speleomantes* to be refractory to *Bd* infection and thus resistant to chytridiomycosis. Resistance to chytridiomycosis would at least in part explain the localized persistence of the genus *Speleomantes* in the presence of highly virulent *Bd*GPL strains in Europe. This situation differs markedly from some of the plethodontids in North America that are susceptible to infection and those in the Neotropics that underwent recent and sharp chytridiomycosis driven declines upon the arrival of *Bd* [15], [16], [51]–[55]. While our results are preliminary evidence that the genus *Speleomantes* is a low-risk taxon for decline due to chytridiomycosis, we recommend additional studies that further investigate the risk *Bd* may pose to this unique amphibian taxon.

## Supporting Information

Table S1 **Overview of the sampled** ***Speleomantes*** **species, sampling localities, sample size and sampling dates.** Seconds have been removed from coordinates to prevent illegal collection.
(DOCX)

Table S2 **Overview of the sampled** ***Speleomantes*** **species for collection of skin secretions and respective sampling localities.** Seconds have been removed from coordinates to prevent illegal collection
(DOCX)
