# Supplementary material for: Resistance to Chytridiomycosis in European Plethodontid Salamanders of the Genus Speleomantes
Source: PLoS One. 2013 May 20;8(5):e63639. doi: 10.1371/journal.pone.0063639 (PMC3659026; doi:10.1371/journal.pone.0063639)
Supplement: Table S1 — Overview of the sampled Speleomantes species, sampling localities, sample size and sampling dates. Seconds have been removed from coordinates to prevent illegal collection. (DOCX) [file pone.0063639.s001.docx]

| Species | Coordinates | Sample size | Date |
| --- | --- | --- | --- |
| *S. flavus* | N 40°35'; E 9°39' | 9 | 30/12/2004 |
|  | N 40°31'; E 9°35’ | 3 | 28/12/2011 |
|  | N 40°31'; E 9°35' | 2 | 28/12/2011 |
|  | N 40°35'; E 9°39' | 5 | 28/12/2011 |
|  | N 40°35'; E 9°41' | 3 | 28/12/2011 |
|  | N 40°30'; E 9°32' | 2 | 21/01/2012 |
|  | N 40°28'; E 9°32' | 7 | 21/01/2012 |
|  | N 40°32'; E 9°35' | 4 | 29/12/2010 |
|  | N 40°28'; E 9°34' | 6 | 8/02/2012 |
|  | N 40°28'; E 9°35' | 1 | 8/02/2012 |
|  | N 40°34'; E 9°41' | 1 | 8/02/2012 |
|  | N 40°35'; E 9°41' | 26 | 8/02/2012 |
| Subtotal |  | 69 |  |
| *S. genei* | N 39°26'; E 8°29' | 5 | 29/12/2011 |
|  | N 39°20'; E 8°38’ | 7 | 29/12/2011 |
|  | N 39°25'; E 8°29' | 29 | 29/12/2011 |
|  | N 39°17'; E 8°28' | 3 | 30/12/2011 |
|  | N 39°12'; E 8°31' | 1 | 30/12/2011 |
|  | N 39°11'; E 8°56' | 6 | 1/01/2012 |
|  | N 39°08'; E 8°44' | 20 | 22/01/2012 |
|  | N 39°20'; E 8°38' | 96 | 22/01/2012 |
|  | N 39°13'; E 8°33' | 6 | 22/01/2012 |
|  | N 39°08'; E 8°40' | 20 | 11/02/2012 |
|  | N 39°12'; E 8°31' | 1 | 11/02/2012 |
| Subtotal |  | 194 |  |
| *S. imperialis* | N 40°03'; E 8°52' | 25 | 4/12/2009 |
|  | N 40°03'; E 8°52' | 9 | 26/12/2011 |
|  | N 40°03'; E 8°52' | 3 | 27/12/2011 |
|  | N 39°49'; E 9°29' | 6 | 31/12/2011 |
|  | N 39°51’; E 9°27' | 1 | 31/12/2011 |
|  | N 39°30’; E 9°24' | 58 | 1/01/2012 |
|  | N 39°53'; E 8°58' | 3 | 21/01/2012 |
|  | N 39°32'; E 9°36’ | 9 | 6/02/2012 |
|  | N 40°08'; E 8°59' | 12 | 9/02/2012 |
|  | N 40°05'; E 8°54' | 1 | 9/02/2012 |
|  | N 40°06'; E 8°54' | 10 | 9/02/2012 |
|  | N 40°03'; E 8°53' | 7 | 9/02/2012 |
|  | N 39°55'; E 8°54' | 5 | 10/02/2012 |
| Subtotal |  | 149 |  |
| *S. sarrabusensis* | N 39°15'; E 9°21' | 2 | 28/12/2004 |
|  | N 39°18'; E 9°27' | 62 | 14/05/2007 |
|  | N 39°15'; E 9°23' | 5 | 31/12/2011 |
|  | N 39°15'; E 9°21' | 1 | 12/02/2012 |
| Subtotal |  | 70 |  |
| *S. supramontis* | N 40°16'; E 9°25' | 4 | 5/12/2009 |
|  | N 40°17'; E 9°30' | 1 | 27/12/2011 |
|  | N 40°17'; E 9°30' | 5 | 27/12/2011 |
|  | N 40°17'; E 9°30' | 5 | 27/12/2011 |
|  | N 40°17'; E 9°30' | 5 | 27/12/2011 |
|  | N 40°18'; E 9°33' | 22 | 29/12/2011 |
|  | N 40°01'; E 9°41' | 14 | 7/02/2012 |
|  | N 40°03'; E 9°40' | 24 | 7/02/2012 |
|  | N 40°19'; E 9°36' | 8 | 7/02/2012 |
| Subtotal |  | 88 |  |
| *S. strinatii* | N 44°38'; E 8°45' | 20 | 4/10/2011 |
|  | N 44°33'; E 8°59' | 20 | 6/10/2011 |
|  | N 44°17'; E 7°26' | 8 | 9/08/2011 |
|  | N 44°09'; E 8°17' | 20 | 8/10/2011 |
|  | N 44°27'; E 9°05' | 22 | 9/11/2011 |
|  | N 43°57''; E 7°36'' | 21 | 31/05/2012 |
|  | N 44°13'; E 8°14’ | 7 | 30/07/2012 |
|  | N 43°44'; E 7°23' | 17 | 30/08/2012 |
| Subtotal |  | 135 |  |
| *S. ambrosii* | N 44°13'; E 9°42' | 22 | 12/10/2011 |
|  | N 44°11'; E 9°43' | 16 | 12/10/2011 |
|  | N 44°07'; E 9°46' | 30 | 18/07/2012 |
| Subtotal |  | 68 |  |
| *S. italicus* | N 44°02'; E 10°18' | 13 | 2/07/2012 |
|  | N 44°02'; E 10°18' | 10 | 2/07/2012 |
|  | N 44°01'; E 10°17' | 3 | 2/07/2012 |
|  | N 44°03'; E 10°18' | 12 | 2/07/2012 |
|  | N 44°02'; E 10°15' | 26 | 20/08/2012 |
|  | N 43°55'; E 11°08' | 28 | 25/08/2012 |
|  | N 43°58'; E 12°27' | 30 | 15/09/2012 |
|  | N 43°24'; E 12°57' | 6 | 23/09/2012 |
|  | N 43°13'; E 12°55' | 20 | 23/09/2012 |
| Subtotal |  | 148 |  |
| **Total** |  | **921** |  |
