# Supplementary material for: Resistance to Chytridiomycosis in European Plethodontid Salamanders of the Genus Speleomantes
Source: PLoS One. 2013 May 20;8(5):e63639. doi: 10.1371/journal.pone.0063639 (PMC3659026; doi:10.1371/journal.pone.0063639)
Supplement: Table S2 — Overview of the sampled Speleomantes species for collection of skin secretions and respective sampling localities. Seconds have been removed from coordinates to prevent illegal collection (DOCX) [file pone.0063639.s002.docx]

| **Species** | **Locality** |
| --- | --- |
| *S. ambrosii* | N 44°11’;E 9°43’ |
| *S. italicus* | N 43°60’;E 10°18’ |
| *S. strinatii* | N 44°33’;E 8°58’ |
| *S. genei* | N 39°08’;E 8°44’ |
| *S. supramontis* | N 40°18’;E 9°33’ |
| *S. flavus* | N 40°28’;E 9°32’ |
| *S. sarrabusensis* | N 39°18’;E 9°27’ |
